# Quality Quantification and Control via Novel Self-Growing Process-Quality Model of Parts Fabricated by LPBF Process

**DOI:** 10.3390/ma15238520

**Published:** 2022-11-29

**Authors:** Xinyi Xiao, Beibei Chu, Zhengyan Zhang

**Affiliations:** 1 Mechanical and Manufacturing Engineering Department, Miami University, Oxford, OH 45056, USA; 2 School of Mechanical Engineering, Hebei University of Technology, Tianjin 300130, China

**Keywords:** self-growing, process-quality model, machine learning, tensile strength, 316L stainless-steel, LPBF

## Abstract

Laser Powder Bed Fusion (LPBF) presents a more extensive allowable design complexity and manufacturability compared with the traditional manufacturing processes by depositing materials in a layer-wised manner. However, the process variability in the LPBF process induces quality uncertainty and inconsistency. Specifically, the mechanical properties, e.g., tensile strength, are hard to be predicted and controlled in the LPBF process. Much research has recently been reported exploring the qualitative influence of single/two process parameters on tensile strength. In fact, mechanical properties are comprehensively affected by multiple correlated process parameters with unclear and complex interactions. Thus, the study on the quantitative process-quality model of the metal LPBF process is urgently needed to provide an enough-strength component via the metal LPBF process. Recent progress in artificial intelligence (AI) and machine learning (ML) provides new insight into quality prediction in terms of computational accuracy and speed. However, the predictive model quality through the traditional AL/ML is heavily determined by the training data size, and the experimental analysis can be expansive on LPBF. This paper explores the comprehensive effect of the tensile strength of 316L stainless-steel parts on LPBF and proposes a valid quantitative predictive model through a novel self-growing machine-learning framework. The self-growing framework can autonomously expand and classify the growing dataset to provide a high-accuracy prediction with fewer input data. To verify this predictive model of tensile strength, specimens manufactured by the LPBF process with different group process parameters (laser power, scanning speed, and hatch spacing) are collected. The experimental results validate the predicted tensile strengths within a less than 3% deviation.

## 1. Introduction

Laser Powder Bed Fusion (LPBF) is a material accumulation process by selectively melting and solidifying metal powders to form three-dimensional objects in a layer-wised manner, contrasting with the traditional subtractive manufacturing processes. This process is capable of handling a large variety of materials and with a much larger design space compared with subtractive manufacturing. However, this process involves repeated rapid heating and cooling during the melting and solidifying of the metal powders processes. Thus, this process comes with high process variability and low repeatability compared with traditional manufacturing processes [1]. Therefore, it is always necessary to conduct destructive/non-destructive qualification methods to measure the qualities of LPBF-ed parts, such as porosity, tensile strength, and fatigue. In addition, post-processing techniques, for example, heat treatment and machining techniques, are always subsequent to the LPBF process to further improve the part finish and enhance the as-built mechanical properties.

**Figure 1:**
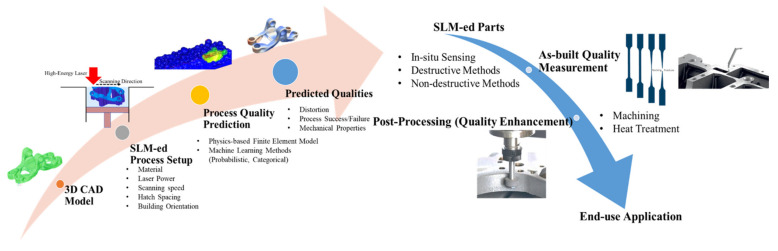
LPBF-ed Part Quality Prediction-Measurement-Enhancement Flow.

Figure 1 indicates the workflow from a digital 3D model to an end-use part produced through the LPBF manufacturing process. This process can be separated into prediction and qualification sections. The process quality prediction involves the consideration of all potential influential parameters’ effects on the as-built qualities. And the quality quantification process heavily relies on the measurement systems, through either monitoring or post-measurements. The process involves extensive human-to-machine interactions and high process uncertainties. Thus, repeatability and reproducibility cannot be ensured.

The typical workflow to predict, measure, and enhance the LPBF-ed components can be seen in Figure 1. To fulfill the end-use requirements, every LPBF-ed part must be verified and validated in its as-built quality. If the parts fail to pass the as-desired requirement, the part needs to be re-printed or processed through certain quality enhancement post-processing techniques. Such an iterative process increases the production cost and lowers the producibility.

Past research mainly focuses on process-quality prediction, which lies in thermal distortion [2] and mechanical properties, such as tensile strength [3] and porosity [4]. However, these studies have the following limitations:

- Physical-based finite element models for distortion prediction vary from software to software and always require high computational time;
- Most probabilistic and categorical prediction models require heavy amounts of training data to increase the accuracy of the model;
- The location of the differently labeled training data, i.e., the distance from the labeled data to the decision boundary, would make it quite difficult for neural networks to make correct classification or prediction.

**Figure 2:**
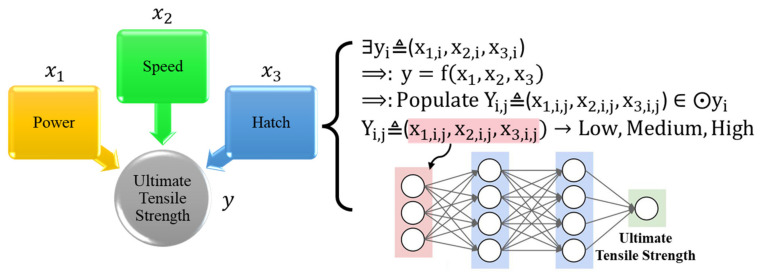
Process-Quality Framework Overall Workflow.

The scope of the previous research [1,2,3,4,5] is limited to exploring the mechanical properties affected by single/two specific process parameters, such as laser energy and layer thickness. However, mechanical properties are comprehensive performance from the LPBF process and are heavily affected by multiple process parameters but with unclear and complex interactions with each other. Thus, a comprehensive quantitative predictive model for analyzing the mechanical properties of the part before the real printing process is urgently needed to ensure component functionality. For example, suppose the predictive model of mechanical properties affected by multiple process parameters comprehensively can be established. In that case, the mechanical properties of the LPBF parts can be predicted and ensured, and the optimal process parameters to fabricate parts with higher performance can be obtained based on this predictive model. The proposed process-quality framework overall workflow is shown in Figure 2.

Experimental data are collected to determine the correct relationship between the process and the ultimate property. However, if the training dataset is small, the machine learning prediction model cannot provide accurate insight to analyze and control the process repeatability quantitatively. Therefore, the self-growing algorithm (Section 4) will populate and self-classify these data. Then these data will be used for developing the predictive model.

In conclusion, this paper presents a comprehensive study on the as-built ultimate tensile strength of 316L stainless steel caused and controlled by the LPBF process. The predictive model contains three key parameters (laser power, scanning speed, and hatch spacing) with intercorrelated effects on tensile strength. In addition, experimental data are collected to verify the effectiveness and accuracy of the established self-growing predictive model. The proposed approach is also applicable to establish predictive models for other as-built properties that are correlated with the multiple process parameters.

## 2. Literature Review

LPBF process is capable of fabricating a large variety of materials, such as steel 300 [5], Ti-6Al-4V [6,7,8], Al-Si10-Mg [9], CoCr alloy [10], Inconel 718 [11], 304 stainless steel [12], and 316L stainless steel [13] is one of the common-use materials for discussing its as-built properties. In this section, we conduct a thorough literature review on the existing AM-ed components’ quality analysis based on experimental and computational analysis.

Numerous studies focus on the SS316L as-built properties in the LPBF process [13,14,15,16,17,18]. Specifically, the surface finish has been mostly discussed [15]. For example, Wang used the experimental method to analyze the surface roughness [16], and Yusuf analyzed the porosity-microhardness relationship to study the microhardness [17]. In addition, Lin analyzed the effects of plasma co-alloying treatments to conduct surface modification [18] on LPBF-ed parts. Besides the surface character, overall thermal distortion has been widely studied and commonly used before the experiment to prevent build failure. Commercial software [19,20,21] uses voxels as the simulation base, and other research works focus on developing accurate laser models and improving computational accuracy and speed [22,23,24]. However, the mechanical properties are the crucial factors to ensure the functionality of the build besides the shape of the products. Recently, many researchers have studied mechanical properties [25,26] and the optimization/control from the process parameters, such as process time interval and heat treatment [27], manufacturing parameters [28,29,30], the particle size distribution effects [31], layer thickness [32], energy input [33], laser parameters [34,35,36,37,38], and environment variables [39]. Specifically, the qualitative relationship between one/two process parameters with one mechanical property has been widely reported. For example, Leicht et al. [40] concluded that higher energy would result in a higher density component. Wang et al. [41] reported a proportional linear relationship between the grain diameter in the direction of applied tensile load and the yield strength. Cherry et al. [42] investigated the inverse proportional relationship between the as-built porosity and the hardness level. In addition, Liverani et al. [43] provided an optimal processing zone with laser power and hatch spacing to guarantee a density greater than 98%. Meier et al. [44] found a dependent relationship between the density and the tensile strength of the SS-316L through experiments.

Besides the abovementioned experimental analysis for developing a qualitative relationship between the process and the desired quality, other researchers used the data analytical methods [45], and machine learning model [46,47,48] to provide a probabilistic predictive model for providing guidance in assuring the as-built quality in the pre-processing stage. However, few researchers developed a novel multi-dimensional process-quality framework [49,50,51,52,53] that can provide a quantitative relation between the multiple process parameter and the build quality and provide a certain printable zone to control the quality as desired.

## 3. Experimental Setup

The 316L stainless-steel powder was selected as the experimental material purchased from the Germany TLS company. The chemical composition is shown in Table 1, and the average particle size was 30 μm.

**Figure 3:**
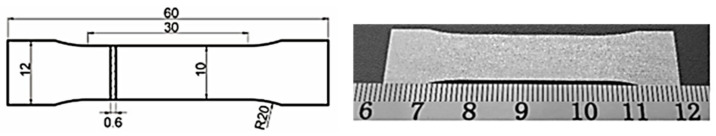
(**left**) Tensile Test Specimen Dimensions, (**right**) the as-built part.

The BLT S200 is adopted in this experiment with a maximum building space of 105 mm × 105 mm × 200 mm, and a 500-W fiber laser was used for vibrating-mirror laser scanning with a wavelength of 1070 mm. The forming substrate was 304 stainless steel. Before the experiment, the surface of the substrate was pretreated with industrial alcohol to ensure that the forming process was not affected by surface oil or dust. Before processing, the substrate was preheated to 80 °C. Argon was used as a protective gas in the molding process, and the oxygen content was kept below 800 ppm. Tensile test pieces were designed according to GB/T 228.1–2010, as shown in Figure 3. Three key process parameters (laser power-P, scanning speed-V, and hatch scan-D) were selected to explore the comprehensive predictive model of tensile strength. The process parameters experimental setup was designed for analyzing the correlated effects towards tensile strength, as shown in Table 2. The tensile test procedure follows the ASTM E8 specification [54]. All specimens are pulled at a strain rate of 0.005in/in/min.

## 4. Proposed Methodology

This research proposes a novel self-growing dynamic neural network framework to establish the comprehensive process-quality predictive model of the LPBF process. One of the main limitations of applying traditional neural networks to additive manufacturing data is the shortage of the dataset. The other drawback of neural networks is over-parameterized neurons and synapses. To overcome such limitations, a point-wise population and self-growing dynamic interference training techniques are developed in this paper without sacrificing the desired task performance (e.g., classification accuracy or prediction quality).

**Figure 4:**
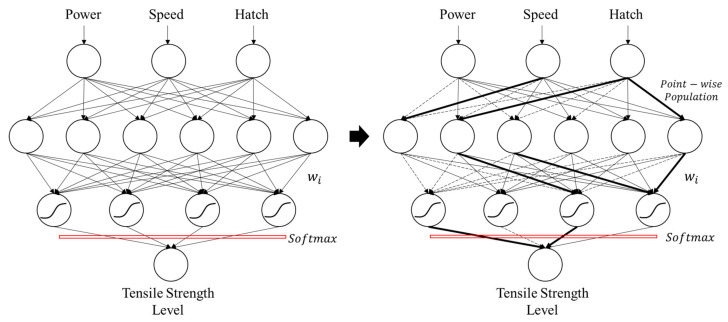
Self-growing Dynamic Neural Network.

The point-wise population is developed to overcome the limitations of the shortage of the dataset. The surface response models based on the collected data are first established. In addition, point-wise population data volumes are created through the constructed response models. These data volumes are designed to adapt to the significance level of the parameter. And the density of the data in the volume is varied based on the level of the tensile strength. The self-growing dynamic interference training schemes are based on easy and hard data. The easy data refers to the data that is located far from the hyperplane, in contrast to the hard data, which sits close to the decision boundary. To leverage the existence of the easy and hard data to accelerate the tensile strength classification within the process-quality model, the highly correlated synapses for the easy data are collected (marked in bold in Figure 4). These synapses are emphasized while processing hard data through the front-end part of the neural network. Similarly, the less-relevant synapses from the easy data are gathered (marked in dashed in Figure 3). These synapses are ignored when processing hard data.

**Figure 5:**
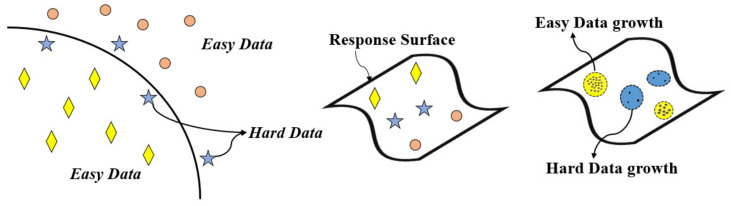
Point-wise population based on the easy/hard data.

Figure 4 presents the self-growing dynamic neural network scheme, which first takes the input parameters to the hidden sigmoid layers, then processes with SoftMax output neurons to predict the level of tensile strength. The key idea of the self-growing training scheme is based on the phenomenon of easy and hard data (as shown in Figure 5). Each data has different locations to the hyperplane/decision boundary in the entire dataset and the constructed surface response model. The closer ones are called hard data, since it is quite hard for the neural network to classify and distinguish them. In contrast, the easy data are far from the decision boundary, making the network easier to classify and recognize. The self-growing scheme heavily depends on the easy/hard data phenomena in training/validating datasets. Through the proposed algorithm, the populated data on the easy ones will be denser and with a larger tolerance zone. In comparison, the self-generated data on the hard ones will be sparser and with a smaller defined zone of collection.

Aiming at the three factors, laser power *P*, scanning speed *V*, and hatch scan *D*, the response model can be formulated as:

(1) $$y=b_{0}+b_{1}P+b_{2}V+b_{3}D+b_{11}P^{2}+b_{22}V^{2}+b_{33}D^{2}+b_{12}PV+b_{13}PD+b_{23}VD+\varepsilon$$

The total effect of the fitting is as follows: R-Sq is close to R-Sq (adj), the fitting effect of the model is better, and R-Sq (pred) is close to R-Sq, which shows that the prediction of the model is reliable. The ANOVA table is presented in Table 3.

The fitted model has the following: R-Sq = 90.86%, R-Sq (pred) = 78.11%, and R-Sq (adj) = 82.63%, with the probability values of P, V, D, P*P, V*V, P*D, and V*D, which are less than the significance level of 0.05, indicating that these effects are all significant. In contrast, the corresponding probability values (W) of the interaction effects of D*D and P*V are 0.148 and 0.144, respectively, greater than the significance level, and the effect is insignificant.

**Figure 6:**
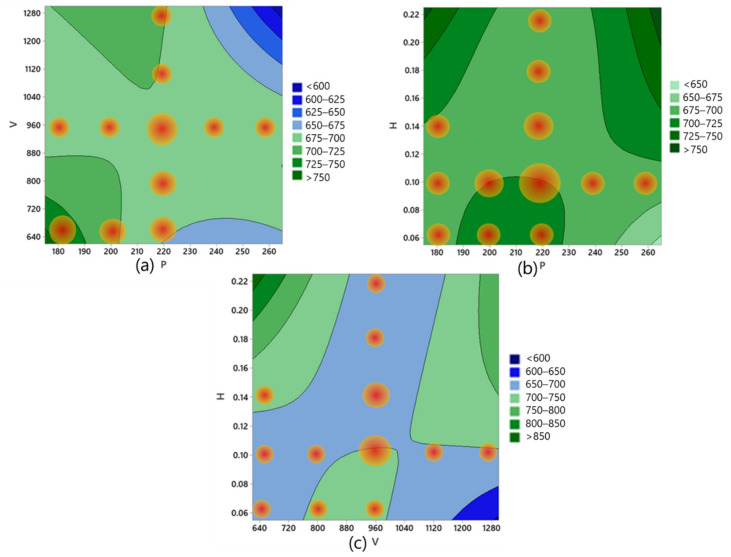
(**a**) Self-growing populated data volume in the P–V–TS model, (**b**) self-growing populated data volume in the P–H–TS model, and (**c**) self-growing populated data volume in the V–H–TS model.

Based on the fitted model, the self-growing zone on the P–V, P–H, and V–H response model based on the classified easy/hard data is indicated in Figure 6. The size and the gradient of the populated data volumes are varied based on the significance of the process parameter and the deviation between the fitted model and the input data.

After obtaining the self-populated data, 80% of the data are used for training and 20% for testing in the following analysis. The accuracy and validation of the proposed machine-learning network will be presented in Section 5.

## 5. Results and Discussions

**Figure 7:**
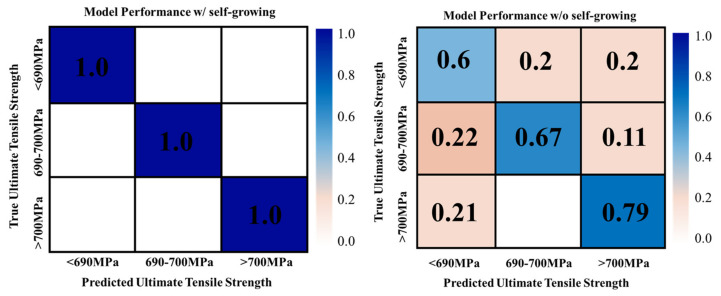
Machine Learning Models Performance.

The self-growing machine learning model performance is shown in Figure 7, comparing the model without the self-growing feature.

The tensile strength has been categorized as low, medium, and high, and the diagonal cells in the matrixes represent the percentage of the correct response level prediction. The higher the number represents that the model is accurate. Furthermore, the cells that are not lying on the diagonal in the matrixes show incorrect classification. Figure 6 shows that the proposed self-growing model presents a 100% accurate prediction for levels 0, 1, and 2. In contrast, the traditional machine learning model presents the highest accuracy of 80%. There is a significant increase in the prediction by adding the self-growing feature to the model.

**Figure 8:**
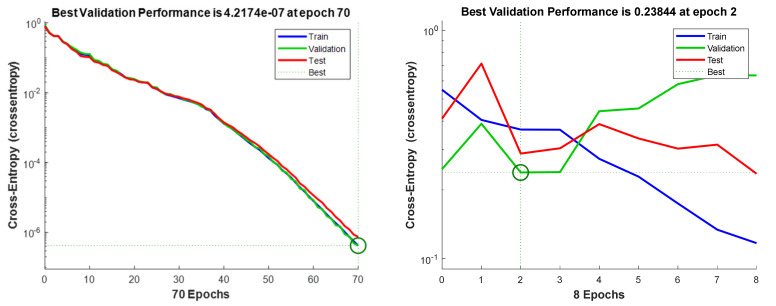
Neural network convergence for Tensile Strength (**left**) with self-growing and (**right**) without self-growing.

The convergence of the neural network is the process by which the network gradually adjusts its weights and biases so that the output of the network is closer and closer to the desired target values. This is conducted through a process of iteration, in which the network is repeatedly exposed to new training data, and the weights and biases are adjusted accordingly. The convergence of the neural network is a key factor in determining the accuracy of the network’s predictions. The convergence of the one with and without the proposed self-growing neural network is presented in Figure 8.

Cross-entropy is a measure of how close a neural network is to convergence. It is calculated by taking the sum of the products of the weights and the errors for each neuron in the network. This measure determines when a neural network has reached a stable state. For example, from the neural network models’ convergence plots in Figure 8, the model with the self-growing function can reach a lower cross-entropy, which means that the network can learn the tensile strength more accurately. This is achieved by ensuring that the network is properly configured and has enough training data provided autonomously through the self-growing function.

Five groups of specimens that varied three analyzed parameters (P, V, D) were fabricated and tested further to verify the correctness of the proposed machine learning model. The test value and calculated values based on the comprehensive predictive model using the self-growing feature are shown in Table 4. In addition, the prediction levels of the tensile without using the self-growing technique are also presented to be used as the comparison group. The categorical level predictions are also presented in the table which are shown in the bracket () manner.

The tensile strength prediction through the proposed machine learning model matches the experimental measures, which indicates the credibility of the prediction model. In addition, from Table 3, the ultimate tensile strength prediction by comparing the two machine learning models has a significant difference, specifically for predicting the high tensile strength. Therefore, the presented self-growing technique can significantly improve prediction accuracy.

The topography of the fracture analysis was measured over the entire area of the test specimen with an optical three-dimensional surface measurement system. Three representative groups of specimens are selected from Table 2 with the representative tensile strength level (low, medium, high), as shown in Table 5.

**Figure 9:**
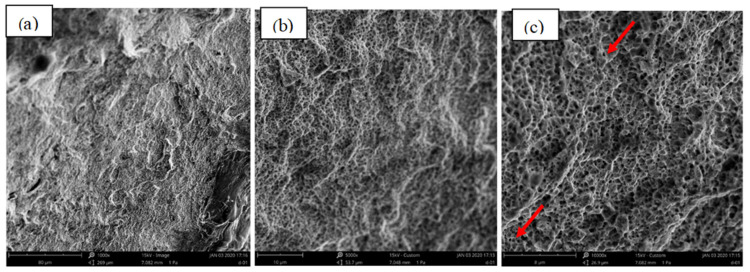
The fracture morphology of group 8—Medium Tensile Strength in Table 4, (**a**) ×1000, (**b**) ×5000, (**c**) ×10,000 (red arrow represents the micro-holes).

**Figure 10:**
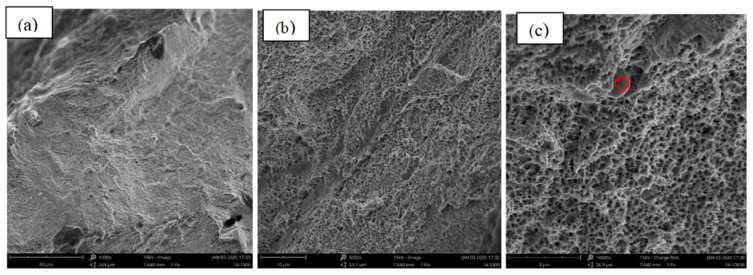
The fracture morphology of group 14—Low Tensile Strength in Table 4, (**a**) ×1000, (**b**) ×5000, (**c**) ×10,000 (circled area represents the EDS performed zone).

**Figure 11:**
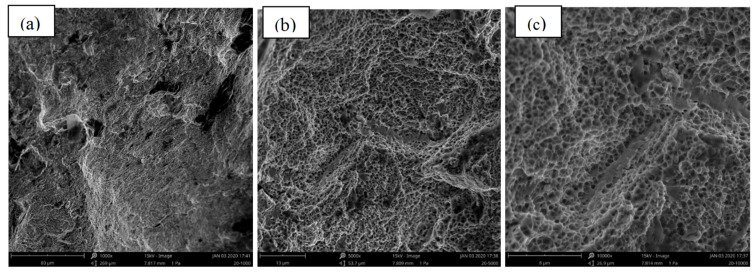
The fracture morphology of group 20—High Tensile Strength in Table 4, (**a**) ×1000, (**b**) ×5000, (**c**) ×10,000.

**Figure 12:**
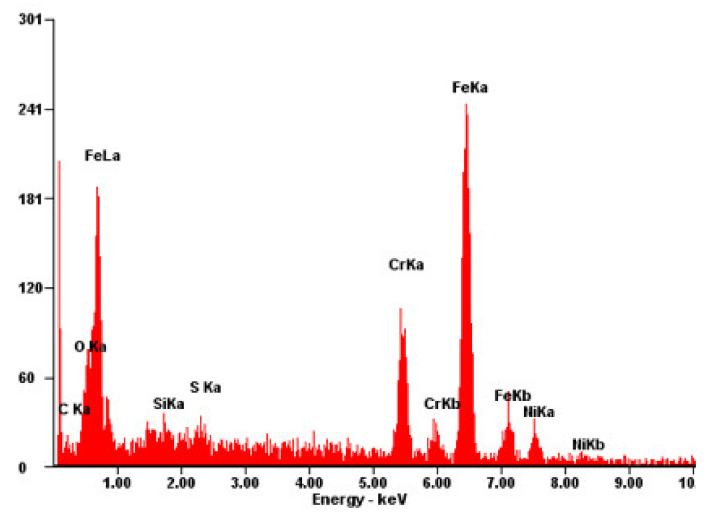
The EDS analysis result for the area that is indicated in Figure 10c.

Figure 9, Figure 10 and Figure 11 present the fracture morphology of the selected three groups of tensile specimens, indicating that the tensile specimens are all ductile fractures with numerous dimples. As shown in Figure 9, it is observed that the dimples are non-uniform, and their size and depth are bigger than the ones that are shown in Figure 9, but smaller than that in Figure 11. In addition, some defects exist, such as the micro-holes (as shown by the red arrows in Figure 10c) intermingled with small dimples and showing holes in some local regions. These fractures with obvious fluctuation, pure shear stress, and crack propagation along the direction of principal stress are characterized by serpentine glide, a typical ductile fracture of dimple micro-groove. Figure 9 shows that it is fractured along the grain boundary, and the dimples with uniform shape and size are smaller than those in Figure 9 and Figure 11. The dimples are shallow in-depth, and small edges with different orientations can be seen on the fracture surface, which is composed of a large number of dimples, and there will be a particle at the bottom of the dimples. Energy-dispersive X-ray spectroscopy (EDS) is performed to analyze the chemical characterization/elemental analysis of the particles indicated in Figure 10c. The obtained results are shown in Figure 12. The outlined areas indicate the un-melted SS316L particles. The un-melted SS316L will reduce the tensile strength. This further verifies the tensile strength test results.

Furthermore, the stress state formed by equiaxial dimples is a uniform strain. From Figure 11, the fracture behavior is a ductile fracture, which illustrates the phenomenon of corner fractures compared to the specimens shown in Figure 8 and Figure 9. This indicates that this group requires a higher strength to break, confirming the data obtained in Table 4. A radial herringbone ridge pattern also appears when the scale is larger. The dimple is elongated parallel to the fracture direction, which generates a ductile sliding stress state.

The fracture morphology and the EDS analysis match the measurement and prediction results. This further reinforces the accuracy of the process-quality prediction model.

## 6. Conclusions

Tensile strength is one of the most important mechanical properties for ensuring additive manufacturing parts’ functionality. However, it is comprehensively affected by multiple process parameters rather than single process parameters. Therefore, selecting the optimal processing condition to fabricate the parts with the desired high performance is difficult since the comprehensive effect is very complicated. Typically, the process parameters behave highly correlated but with unclear and complex interactions with each other. Thus, an accurate and computationally efficient tensile strength predictive model by multiple process parameters is desired and significant to assist when selecting the optimal processing condition. The following qualitative conclusions can be obtained from the proposed research to achieve a higher tensile strength component:

- An increase in power should be coupled with a higher scanning velocity;
- A smaller hatch spacing would be set with a higher laser power;
- Velocity and the hatch spacing should be inverse proportional relation.

The research of this paper focuses on comprehensively analyzing the tensile strength 316L stainless-steel parts and establishing a self-growing machine learning-based predictive model with three key parameters (laser power, scanning speed, and hatch scan). The experimental tensile test results and the fracture morphology both indicate that this proposed predictive model and its corresponding establishment approach can explicitly predict the ultimate tensile strength and establish an optimal printable zone that ensures the high-end components of the LPBF process. This research paves the path for building a digital twin on the metal AM process from design, material, process, and quality in an effective manner.

## Figures and Tables

**Table 1:** Chemical composition of the 316L stainless steel.

Chemical Composition	Ni	Cr	Mn	Si	Mo	C	P	S	Cu
Percentage (%)	10.76	16.78	1.23	0.58	2.42	0.018	0.011	0.007	0.08

**Table 2:** The Experimental Setup.

Process Parameters	Experimental Range
P (W)	[160, 280]
V (mm/s)	[800, 1200]
D (mm)	[0.06, 0.22]

**Table 3:** The ANOVA table that displays the results of a statistical analysis of variance.

	Freedom	Seq SS	Adj SS	Adj MS	F	W
Regression	9	989.22	989.22	109.914	11.04	0.000
Linear	3	209.47	482.54	160.847	16.16	0.000
P	1	0.24	106.93	106.926	10.74	0.008
V	1	33.33	204.84	204.842	20.58	0.001
D	1	175.90	470.83	470.828	47.31	0.000
Square	3	611.10	346.07	115.356	11.59	0.001
P*P	1	366.22	326.59	326.594	32.81	0.000
V*V	1	182.25	132.32	132.319	13.29	0.004
D*D	1	62.63	24.40	24.403	2.45	0.148
Interaction	3	168.65	168.65	56.216	5.65	0.016
P*V	1	69.23	25.03	25.026	2.51	0.144
P*D	1	8.77	56.62	56.616	5.69	0.038
V*D	1	90.65	90.65	90.652	9.11	0.013
Residual Error	10	99.53	99.53	9.953		
Misuse	7	85.42	85.42	12.203	2.60	0.233
Pure error	3	14.11	14.11	4.702		
Total	19	1088.75				
S = 3.15478 PRESS = 499.659 R-Sq = 90.86% R-Sq (pred) = 78.11% R-Sq (adj) = 82.63%						

**Table 4:** The Validation Experimental Setup.

Experimental Data (P, V, H)	Predict Level (Without Self-Growing)	Predicted TS (MPa) (With Self-Growing)	Measured TS (MPa)
(220,960,0.10)	2	700.284 (2)	705.076 (2)
(220,900,0.10)	1	700.398 (2)	701.782 (2)
(220,960,0.15)	0	689.849 (1)	693.550 (1)
(240,1120,0.10)	1	695.245 (1)	697.371 (1)
(240,960,0.06)	0	712.138 (2)	713.792 (2)

**Table 5:** Selected groups of specimens for fracture analysis.

Group Num.	P (W)	V (mm/s)	D (mm)	Measured UTS (MPa)	Level
8	220	1120	0.1	693.691	Medium
14	180	640	0.06	682.111	Low
20	220	960	0.1	705.076	High
